# 

**Published:** 1892-01

**Authors:** 


					﻿Entered at the Post Office, at Austin, Texas, as Second Class Mail Matter
VOL. VII.
JANUARY, 1892,
No. 7.
DANIEL'S
MEDICAL
JOURNAL
Subscription, $2.00 a Year, in Advance. – Single Copies, Twenty-five Cents.
F. E. DANIEL., M. D., Editor and Publisher, AUSTIN, TEXAS.
Eugene Von Boeckmann, Steam Book and Jor Printer, Austin, Texas
				

